# Household air pollution and childhood stunting in China: A prospective cohort study

**DOI:** 10.3389/fpubh.2022.985786

**Published:** 2022-10-21

**Authors:** Min Yao, Lingou Li, Mei Yang, Yuanyuan Wu, Feifei Cheng

**Affiliations:** ^1^Research Center for Economy of Upper Reaches of the Yangtze River, Chongqing Technology and Business University, Chongqing, China; ^2^Department of Endocrinology, The First People's Hospital of Chong Qing Liang Jiang New Area, Chongqing, China; ^3^Health Management Center, The Second Affiliated Hospital of Chongqing Medical University, Chongqing Medical University, Chongqing, China

**Keywords:** household air pollution, solid fuel, childhood stunting, socioeconomic indicators, mediation analysis, China

## Abstract

**Background:**

Exposure to air pollution, especially indoor air pollution, was associated with an increased risk of childhood stunting. However, few longitudinal studies have explored the long-term impacts of indoor air pollution from household solid fuel use on child growth. We aimed to investigate the association between household air pollution (HAP) from solid fuel use and childhood stunting in Chinese children.

**Method:**

The longitudinal data from the Chinese Family Panel Study over 2010–2018 were included in this study with a total of 6,013 children aged 0–15 years enrolled at baseline. Exposure to HAP was measured as solid fuel use for cooking, while solid fuel was defined as coal and firewood/straw according to the questionnaire survey. Stunting was defined as−2SD below the height-for-age z-score (HAZ) of the reference children. Logistic regression and Cox proportional hazards models with time-varying exposures were employed to estimate the association between childhood stunting and HAP exposure.

**Results:**

At baseline, children with exposure to HAP from combusting solid fuels had a relatively higher risk of stunting [OR (95%CI): 1.42 (1.24–1.63)]. Among children without stunning at baseline, those living in households with solid fuel use had a higher stunting risk over an 8-year follow-up [HR (95%CI): 2.05 (1.64–2.57)]. The risk of childhood stunting was increased for those with HAP exposure from firewood/straw combustion or with longer exposure duration [HR (95%CI): 2.21 (1.74–2.79) and 3.01 (2.23–4.08), respectively]. Meanwhile, this risk was significantly decreased among children from households switching from solid fuels to clean fuels [HR (95%CI): 0.53 (0.39–0.70)]. Solid fuel use was suggested to be a mediator of the relationship between poor socioeconomic factors (i.e., household income and parental education level) and childhood stunning, with a mediation effect ranging from 11.25 to 14.26%.

**Conclusions:**

HAP exposure from solid fuel use was associated with childhood stunting. Poor parental education and low household income might be socioeconomic factors contributing to solid fuel use. Therefore, household energy policies to facilitate access to clean fuels are urgently needed, especially for low-income and low-educated households.

## Introduction

Childhood stunting remains a major public health concern over the world. Stunting in children is associated with childhood infection, mortality, impaired cognitive function and educational performance (1–3). In the long term, growth retardation during childhood can lead to irreversible adverse effects on height, increased risk of nutrition-related chronic diseases and poor economic conditions in adulthood (4–8). Although the global prevalence of childhood stunting has declined steadily since 2000 (9), it is still unacceptably high in some economically underdeveloped countries (10). Previous stunting studies mainly focused on the effects of prenatal maternal exposure on fetal outcomes and conditions associated with early infant development, like recurrent infections, chronic diseases, genetic or congenital factors, and dietary nutritional deficiencies (4, 11), and only until recently have the impacts of postnatal children exposure to air pollution, especially the indoor air pollution, on child growth gained increasing attention from researchers.

Combustion of solid fuels is a major source of indoor air pollution that threatens public health worldwide, and remains widespread in low- and middle-income countries (LMICs) including China (12, 13). As reported (14), 41% of households globally (over 2.8 billion people) depend on solid fuel use to meet their basic energy needs for cooking and heating, with children of these households having an increased exposure to household air pollution (HAP) from solid fuels. According to the data from World Health Organization (WHO), nearly 98% of young children aged 5 years and less in LMICs are exposed to excessive air pollution (15, 16). Besides, compared to adults, children have a larger lung surface area per unit of body weight and breathe considerably more air per unit of body weight under normal breathing, resulting in greater exposure to HAP from solid fuels (17, 18). Moreover, they are more susceptible to air pollution than adults given they are at a stage of rapid development of organs and have immature immune systems (19, 20).

It is still inconclusive for the relationship between indoor air pollution due to solid fuels and growth retardation in children. Several cross-sectional studies conducted in LMICs showed that solid fuel use was significantly associated with a delayed child growth and development (21–23). However, a cross-sectional study (*N* = 1150) in Swaziland failed to find any significant association between solid fuel use and stunting in children (24), so did another cross-sectional survey study of multi-country in South Asia (6). Meanwhile, these cross-sectional studies were unable to evaluate the influence of HAP exposure on childhood stunning during follow-up. To the best of our knowledge, there were only two studies of longitudinal design regarding this topic: A recent research using longitudinal data of about 12,000 children from the Young Lives Study found that those living in households using solid fuels were more likely to have a lower height-for-age z-score (HAZ score) (25), however, the researchers did not exclude children with pre-existing stunning and only showed the relationship between fuel use and HAZ score itself, rather than disease status (25); The other study from Nepal with only a one-year follow-up duration reported a negative but not statistically significant relationship between the use of solid fuels and linear growth in children (26). Generally, children involved in the previous studies were relatively young (age < 6 years) and the long-term impact of HAP exposure on child growth still warrant further research. Therefore, a large cohort with satisfactory follow-up duration is urgently needed to address this topic.

Stunting in children was strongly associated with indoor air pollution. However, the relationship between HAP and incident stunting in children has not been examined thoroughly. We aimed to investigate the association between solid fuel use and childhood stunting from both cross-sectional and longitudinal perspectives using a large cohort from China with extensive longitudinal follow-up. In addition, we performed the mediation analysis to evaluate the effect of household fuel use on the association between socioeconomic status (SES) factors and childhood stunting. Finally, we tested the hypothesis that fuel use switching to clean fuel use could reduce the stunting risk among children.

## Materials and methods

### Data source and study sample

China Family Panel Studies (CFPS, http://www.isss.pku.edu.cn/cfps) is a nearly nationwide longitudinal social survey implemented by the Institute of Social Science Survey at Peking University. It surveyed ~14,000 households each year and required all participants to provide written informed consent. Ethical approval for the study was granted by the Ethical Review Committee of Peking University. Five national waves of data are fully available to date (waves in 2010, 2012, 2014, 2016, and 2018) and were included in this study. More detailed information about CFPS is available elsewhere (27).

To investigate both the short- and long-term effects of household fuel use on childhood stunting, participants were selected from the whole cohort as follows: we first excluded children without family and parental information and those without stunting data at baseline, with 6013 children being involved in the cross-sectional analysis. Then, children who were already stunted at baseline or those lacking stunting record(s) in either of the next waves were excluded in the longitudinal analysis. We also dropped children with missing data on household fuel use during follow-up period. Finally, there were 1,789 and 772 children included in the longitudinal analysis for the short-term effect (4 years from 2010 to 2014) and long-term effect (8 years from 2010 to 2018), respectively. The detailed process for participants selection was presented in Supplementary Figure S1, and the provincial distribution of these children was given in Supplementary Figure S2.

### Assessment of stunting in children

The WHO compares the developmental status of children from different countries and regions and provides the international standard for child growth. Using these standards, we calculated the HAZ score for each subjects, which was widely used to assess the children's growth status (28). Stunting was defined as a HAZ below−2 SD of the median for their age and sex, while severe stunting was defined as HAZ below−3 SD of the median (6). For the stability of the model, children with a HAZ score beyond ±7SD were excluded to minimize the influence of extreme values (29).

### Assessment of solid fuel use and clean fuel use

The use of solid fuel related to cooking was determined by a questionnaire conducted with the question: “What kind of fuel does your family normally use for cooking?”. Cooking fuel source was divided into: (1) firewood/straw; (2) coal; (3) gas/liquid; (4) natural gas; (5) solar energy; (6) electricity; (7) others. The use of solid fuel was defined as the primary use of coal or firewood/straw for cooking, while clean fuel use was defined as the primary use of coal gas, liquefied petroleum gas, natural gas, solar energy or electricity for cooking. Clean fuel use also included other fuels because they produced less air pollution than the solid fuels (30). The duration of solid fuel use from 2010 to 2014 was defined as 0, 1–3 years, and 4 years or more, while the duration of solid fuel use between 2010 and 2018 was defined as 0, 1–7 years, and 8 years or more for estimating the short-term and long-term effect of fuel use, respectively.

### Covariates

Based on previous studies (22, 29, 31, 32), we considered several potentially confounding covariates associated with household fuel use and childhood stunting. The potential confounders included age, gender, maternal and paternal education level, maternal and paternal heights, household income, residence, the number of siblings, the classification of cooking water. In addition, variables including annual average PM_2.5_ level (collected from the Atmospheric Composition Analysis Group), province dummy, breastfeeding, and birth weight were further considered in the sensitivity analysis.

### Statistical analysis

Baseline characteristics of the children were presented as median (Q1, Q3), or proportion (%), as appropriate. Student's *t*-test (for continuous variables) and Chi-square test (for categorical variables) were applied to compare the baseline characteristics between children of households with solid fuel use vs. those with clean fuel use.

Logistic regression was employed to investigate the effect of household fuel use on stunting among children at baseline. Besides, in order to control potential selection bias, Propensity score matching (PSM, nearest-neighbor matching with caliper) was used to adjust for the difference in characteristics at baseline (33). Furthermore, Cox proportional hazards regression with time-varying exposures was used to analyze the short- and long-term effects of household fuel use on childhood stunting. The fuel use information in each wave was included for data analysis. The endpoint was defined as the occurrence of stunting. And then, we performed the sensitivity analyses stratified by the residence (urban vs. rural areas).

Afterwards, we performed mediation analyses at baseline to explore the influence of household fuel use on the association between the SES factors and childhood stunting (Supplementary Figure S3), in which the stepwise approach using logistic regression was conducted to test whether solid fuel use played a mediating role between socioeconomic status and childhood stunting. To assess the indirect effect, we also used the bias-corrected bootstrapping (1,000 replications) to calculate the confidence intervals of indirect effect (34). The significant indirect effect was defined as the 95% confidence interval did not include zero (35). In addition, considering that the stepwise approach may miss some potential mediation effects, we used the Sobel test (36) to increase the accuracy. Finally, the effects of households switching to clean fuel on childhood stunting during follow-up were examined. Stata/MP version 17.0 software was used all Statistical analyses. The statistical significance threshold was defined as the *p* < 0.05.

## Results

### Summary of baseline characteristics

The baseline characteristics of the selected children (*N* = 6,013) were shown in Table 1. The median age (Q1-Q3) was 8 (4–12) years old, with 52.4% being boys. More than half (59.8%) of the children lived in rural areas. There were 49.0% of mothers and 61.1% of fathers with education level higher than primary school. Over 85% of households earned more than 10,000 yuan in the latest year. More than half of children [3,150 (52.4%)] were exposed to household solid fuels, with the proportion of 19.7% in urban areas and 80.3% in rural areas. In the longitudinal analysis with 772 children, the percentages of children with newly identified stunting were 20.0% in 2012, 10.4% in 2014, 3.6% in 2016, 1.9% in 2018, and a total of 32.1% (*N* = 248) children had stunted development during 8 years follow-up (Supplementary Table S1). More detailed information about the baseline characteristics of the longitudinal study population was provided in the Supplementary Table S2. The comparison between subjects included and excluded in the cross-sectional and longitudinal analysis were shown in Supplementary Tables S3, S4.

**Table 1:** Baseline characteristics of children grouped by fuel use type in the cross-sectional analysis.

**Characteristics**	**Total**	**Clean fuel**	**Solid fuel**	***P*-value**
	***N* = 6,013**	***N* = 2,863**	***N* = 3,150**	
Age (years)	8.00 [4.00; 12.00]	8.00 [4.00; 12.00]	8.00 [4.00; 12.00]	0.239
Gender				0.673
Boy	3,149 (52.4%)	1,508 (52.7%)	1,641 (52.1%)	
Girl	2,864 (47.6%)	1,355 (47.3%)	1,509 (47.9%)	
Paternal height (meters)	1.70 [1.66; 1.73]	1.70 [1.68; 1.74]	1.70 [1.65; 1.72]	< 0.001
Maternal height (meters)	1.60 [1.56; 1.63]	1.60 [1.56; 1.63]	1.60 [1.55; 1.62]	< 0.001
Paternal education:				< 0.001
Under primary school	2,337 (38.9%)	719 (25.1%)	1,618 (51.4%)	
Above primary school	3,676 (61.1%)	2,144 (74.9%)	1,532 (48.6%)	
Maternal education:				< 0.001
Under primary school	3,066 (51.0%)	965 (33.7%)	2,101 (66.7%)	
Above primary school	2,947 (49.0%)	1,898 (66.3%)	1,049 (33.3%)	
Residence:				< 0.001
Urban	24,18 (40.2%)	1,798 (62.8%)	620 (19.7%)	
Rural	3,595 (59.8%)	1,065 (37.2%)	2,530 (80.3%)	
Household income:				< 0.001
Below 10,000 yuan/year	894 (14.9%)	248 (8.66%)	646 (20.5%)	
Above 10,000 yuan/year	5,119 (85.1%)	2,615 (91.3%)	2,504 (79.5%)	
Siblings:				< 0.001
1	2,880 (47.9%)	1,727 (60.3%)	1,153 (36.6%)	
2	2,321 (38.6%)	901 (31.5%)	1,420 (45.1%)	
≥3	812 (13.5%)	235 (8.21%)	577 (18.3%)	
Cooking water:				< 0.001
Tap/purified water	3,215 (53.5%)	2,163 (75.6%)	1,052 (33.4%)	
Surface-exposed/well water	2,798 (46.5%)	700 (24.4%)	2,098 (66.6%)	

All data are expressed as median [Q1-Q3] or proportion in %; All comparison was conducted between subjects with different fuel use type by using either the Student's t-test for continuous data or the Chi-square test for categorical data.

### HAP exposure from solid fuel use was associated with an increased risk of childhood stunting at baseline in the cross-sectional analysis

Table 2 and Supplementary Table S5 presented the association between HAP exposure from solid fuel use and stunting at baseline among children using unmatched and matched data. All the key assumptions of the PSM were satisfied (Supplementary Table S6, Supplementary Figures S4, S5). Endpoints including both childhood stunting and severe stunting were examined to validate the consistence of results. As shown, the results using unmatched and matched data were generally consistent, suggesting a robust relationship that HAP exposure from solid fuels was associated with a higher risk of childhood stunning at baseline. Compared with households using clean fuels, a significantly increased risk of childhood stunting was observed in households using solid fuels, which remained significant after controlling for potential confounders [OR (95%CI): 1.42 (1.24–1.63), *p* < 0.001]. Different type of solid fuels showed different effects on child growth and development, children with the exposure of firewood/straw had the highest stunting risk than others [OR (95%CI): 1.47 (1.27–1.70), *p* < 0.001].

**Table 2:** Association between exposure to household air pollution from solid fuel use and childhood stunting at baseline using unmatched and matched data.

	**Unmatched data**	**Matched data**
	**OR (95% CI)**	**OR (95% CI)**
**Household fuel use**
Clean fuels	1.00 (ref)	1.00 (ref)
Solid fuels	1.42 (1.24,1.63)***	1.42 (1.22,1.64)***
**Types of household fuel use**
Clean fuels	1.00 (ref)	1.00 (ref)
Coal	1.30 (1.07, 1.58)**	1.27 (1.02, 1.60)*
Firewood/straw	1.47 (1.27, 1.70)***	1.47 (1.25, 1.73)***

All the models were adjusted for age, gender, parental education, parental height, household income, residence, siblings and cooking water; *p < 0.05; **p < 0.01; ***p < 0.001.

Supplementary Table S7 showed the results of sub-group analysis stratified by the residence, with all estimates being consistent with the main results. We also tested the interaction between solid fuel use and residence (rural vs. urban) on childhood stunning in the cross-sectional analysis (*p* = 0.123). Children who exposed to HAP in both rural and urban areas had a higher risk of stunning at baseline, compared with those using clean fuels. In the sensitivity analyses, adjusting for other confounders (Supplementary Table S8), including birth weight, breastfeeding, PM_2.5_, and province dummy, and excluding children with a HAZ score beyond ±6SD (Supplementary Table S9) did not change the relationship between HAP and childhood stunting at baseline.

### HAP exposure from solid fuel use was associated with the incident childhood stunting in the longitudinal analysis

Time-varying Cox model was used to assess the relationship between household fuels use and the future risk of childhood stunting among children without stunting at baseline over a 4- and 8-year follow-up period (Table 3). Compared with households using clean fuels, the short-term HR (95% CI) of solid fuel use on childhood stunting was 1.40 (1.15–1.71) after adjusting for potential confounders, and the long-term HR (95% CI) was 1.46 (1.15–1.86). The effects of different solid fuels on childhood stunning were different. Compared with households using clean fuels, the highest risk of childhood stunting was observed in those households with firewood/straw use [HR (95% CI): 1.41 (1.14–1.73)] in the short-term analysis and 1.52 (1.18–1.96) in the long-term analysis). A positive trend was observed between the duration of solid fuel use and the stunting risk over time. Compared to other groups, children with the longest duration of solid fuels exposure had the highest stunting risk both in the short-term analysis [1.60 (1.23–2.10)] and the long-term analysis [1.77 (1.25–2.52)].

**Table 3:** Hazard ratios of household air pollution for incident childhood stunting in the longitudinal analysis.

**Follow-up period**		**Model 0**	**Model 1**	**Model 2**	**Model 3**
		**HR (95%CI)**	**HR (95%CI)**	**HR (95%CI)**	**HR (95%CI)**
2010 → 2014	
	Household fuel use
	Clean fuels	1.00 (ref)	1.00 (ref)	1.00 (ref)	1.00 (ref)
	Solid fuels	2.05 (1.70, 2.47)***	2.06 (1.72, 2.47)***	1.65 (1.36, 2.01)***	1.40 (1.15, 1.71)***
	Types of household fuel use
	Clean fuels	1.00 (ref)	1.00 (ref)	1.00 (ref)	1.00 (ref)
	Coal	1.61 (1.13, 2.30)**	1.59 (1.11, 2.28)*	1.43 (0.99, 2.05)	1.37 (0.96, 1.97)
	Firewood/straw	2.16 (1.78, 2.62)***	2.18 (1.81, 2.64)***	1.71 (1.39, 2.10)***	1.41 (1.14, 1.73)**
	Duration of solid fuel use
	0	1.00 (ref)	1.00 (ref)	1.00 (ref)	1.00 (ref)
	1–3 years	1.82 (1.43, 2.31)***	1.92 (1.51, 2.43)***	1.55 (1.20, 2.01)***	1.30 (1.00, 1.69)*
	≥4 years	2.67 (2.12, 3.36)***	2.74 (2.18, 3.43)***	2.05 (1.60, 2.64)***	1.60 (1.23, 2.10)***
2010 → 2018	
	Household fuel use
	Clean fuels	1.00 (ref)	1.00 (ref)	1.00 (ref)	1.00 (ref)
	Solid fuels	2.05 (1.64, 2.57)***	2.06 (1.65, 2.58)***	1.78 (1.41, 2.26)***	1.46 (1.15, 1.86)**
	Types of household fuel use
	Clean fuels	1.00 (ref)	1.00 (ref)	1.00 (ref)	1.00 (ref)
	Coal	1.48 (0.94, 2.33)	1.46 (0.92, 2.30)	1.37 (0.87, 2.17)	1.24 (0.79, 1.94)
	Firewood/straw	2.21 (1.74, 2.79)***	2.23 (1.77, 2.81)***	1.90 (1.49, 2.43)***	1.52 (1.18, 1.96)**
	Duration of solid fuel use
	0	1.00 (ref)	1.00 (ref)	1.00 (ref)	1.00 (ref)
	1–7 years	1.70 (1.30,2.22)***	1.78 (1.36,2.33)***	1.50 (1.12,2.01)**	1.20 (0.88,1.63)
	≥8 years	3.01 (2.23, 4.08)***	3.04 (2.25, 4.09)***	2.47 (1.78, 3.42)***	1.77 (1.25, 2.52)**

Model 0: unadjusted; Model 1: adjusted for age and gender; Model 2: model 1 plus adjustment for parental education, parental height; Model 3: model 2 plus adjustment for household income, residence, siblings, cooking water. *p < 0.05; **p < 0.01; ***p < 0.001.

In the sub-group analysis stratified by the residence (Supplementary Table S11), solid fuel use and longer duration was still associated with an increased stunting risk among rural areas. However, the significance in urban areas was attenuated after full adjustment. The *p* values of the interaction between solid fuel use and residence on childhood stunning were both nonsignificant both in the short-term longitudinal analysis (2010–2014) and in the long-term longitudinal analysis (2010–2018) (*p* = 0.913 and 0.188, respectively). In the sensitivity analyses, the relationship between solid fuel use and future risk of stunning remained significant after adjusting for other control variables (Supplementary Table S12) and after excluding those children with HAZ score beyond ±6SD (Supplementary Table S13).

### Mediation analysis

Table 4 presented the results of the mediation effect analysis using the data at baseline, poor SES were found to be associated with a higher stunting risk among children, and they were also the possible contributors for the solid fuel use in households. Additionally, the Z statistics of the Sobel test for household income, parental and maternal education level were 5.20, 5.37, and 5.26, respectively. Based on the 1,000 bootstrap resamples, the 95% bootstrap CI for the indirect effect excluded zero. Therefore, there was a significant mediation effect of solid fuel use between SES and childhood stunning. The proportion of mediation effect of solid fuel use was 14.26% for poorer household income, 14.23% for lower paternal education level and 11.25% for lower maternal education level.

**Table 4:** Mediation effect analysis among socioeconomic status, fuel use and childhood stunning.

**SES (X)**	**Solid fuel use (M)**	**Stunting (Y)**	**Solid fuel use (M)**	**Stunting (Y)**	**Solid fuel use (M)**	**Stunting (Y)**
Household income:						
Above 10,000 yuan/year	1.00 (ref)	1.00 (ref)				
Below 10,000 yuan/year	2.18 (1.83, 2.61) ***	1.50 (1.28, 1.75) ***				
Paternal education:						
Above primary school			1.00 (ref)	1.00 (ref)		
Below primary school			1.90 (1.67, 2.17) ***	1.45 (1.28, 1.64) ***		
Maternal education:						
Above primary school					1.00 (ref)	1.00 (ref)
Below primary school					2.19 (1.92, 2.49) ***	1.72 (1.52, 1.96) ***
Z for Sobel-test	5.20		5.37		5.26	
Mediation effect percentage	14.26%		14.23%		11.25%	

All the model adjusted for age, gender, parental height, residence, siblings, cooking water. ***p < 0.001.

SES, socioeconomic status.

### Switching types of fuel use and childhood stunting

As shown in Table 5, switching from solid fuels to clean fuels was associated with a significantly reduced stunting risk among children (Table 5). Of 772 children included for the longitudinal analysis, 12.3% of them were exposed to solid fuels all the time and 33.9% lived in households switching to clean fuels during follow-up. Compared with those who used solid fuels all the time, those who switched to clean fuels during follow-up had a significantly lower risk of childhood stunting [HR (95% CI): 0.62 (0.47–0.83)], but we did not observe an increased risk of childhood stunting in those who switched from clean fuel use at baseline to solid fuel use during follow-up [HR (95% CI): 0.95 (0.64–1.39)]. Further, we explored the specific effects of when to switch from solid fuels to clean fuels on the risk of stunting and found that the earlier switching to using clean fuels was associated with a lower risk of childhood stunting (OR (95%CI) per 2 years: 0.78 (0.71–0.85), *p* < 0.001, Supplementary Table S14).

**Table 5:** Hazard ratios of switching fuel types for the risk of childhood stunting.

**Group**	**Model 0**	**Model 1**	**Model 2**	**Model 3**
	**HR (95%CI)**	**HR (95%CI)**	**HR (95%CI)**	**HR (95%CI)**
Group 1	1.00 (ref)	1.00 (ref)	1.00 (ref)	1.00 (ref)
Group 2	0.53 (0.39, 0.70)***	0.55 (0.41, 0.73)***	0.56 (0.42, 0.75)***	0.62 (0.47, 0.83)**
Group 3	0.74 (0.49, 1.11)	0.76 (0.52,1.13)	0.83 (0.56,1.23)	0.95 (0.64, 1.39)
Group 4	0.33 (0.25, 0.45)***	0.33 (0.24, 0.44)***	0.41 (0.29, 0.56)***	0.57 (0.40, 0.81)**

Group 1: households using solid fuels all the time; Group 2: households switching to clean fuels during follow-up; Group 3: households switching to solid fuels during follow-up; Group 4: households using clean fuels all the time. Model 0: unadjusted; Model 1: adjusted for age and gender; Model 2: model 1 plus adjustment for parental education, parental height; Model 3: model 2 plus adjustment for household income, residence, siblings, cooking water. **p < 0.01; ***p < 0.001.

## Discussion

In this large cohort study of Chinese children with a moderately long follow-up duration, we demonstrated that HAP exposure from solid fuel use was not only associated with prevalent childhood stunting at baseline, but also closely tied with incident stunting during follow-up. A longer duration of solid fuel use was explicitly linked to a higher risk of stunting, while switching to clean fuel use could significantly reduce the risk of stunting over time. Those observed relationships were independent of traditional risk factors for stunting.

In this study, the adverse effects of HAP on childhood stunting were multidimensionally demonstrated, given the highly consistent results from the cross-sectional and the longitudinal analyses. A study from Bangladesh found that children living in households using biomass fuel were more likely to have underweight and stunting problems (22). Another work from 59 LMICs using half a million analytical sample showed a higher risk of stunting in children exposed to HAP from solid fuels (23). There was only one previous study focused on Chinese children, and it reported that HAP exposure from solid fuels was significantly associated with elevated stunting risks among children aged 6–17 years, especially among girls and high-age children (19). However, those cross-sectional studies have limited ability to investigate the relationship between HAP and future risk of stunning. Since children spend most of the time at home in their early life, and longer duration of HAP with an accumulative exposure might result in a progressively higher risk of growth retardation, the longitudinal effects of HAP on children development need to be further confirmed. Our findings were important complements to previous research in this area.

Our study suggested that the relationship between HAP and childhood stunting in urban and rural areas were generally consistent with the main results both transversely and longitudinally. However, unlike the rural area group, the significance in the urban area group was relatively short of statistical power in the fully adjusted model. Given the higher popularity of solid fuel use in the rural areas, we believe the insignificant results may be due to the relatively lower percentage of solid fuel use and the small sample size after stratification in urban areas. This is also supported by our interaction analysis of solid fuel use and residence which showed no significant difference in the effect size of HAP on childhood stunning between urban and rural areas.

Lower level of maternal education and poorer economic status were strongly associated with developmental delays in children (19, 32). Parents with a higher level of education and income concerned more about the nutritional status of their children (22, 37, 38). Given that factors such as nutrition, diet, breastfeeding, and baby care are especially crucial for child development (32), parents might pay more attention to the above factors. Interestingly, our findings figured out that the choice of fuel type may be another possible mediating factor between the SES and child growth, which might be ignored usually. Consistent with previous studies (39, 40), the present research found that low parental education level and low household income might lead to a greater household preference to solid fuels as an energy source, and the solid fuel use might be a mediating factor between poorer socioeconomic factors and stunting in children. To our knowledge, this is the first study to explore the influence of household fuel use on the association between the SES factors and stunting. What's more, our findings provided a possible mechanism of their mediation, which requires more evidence to further demonstrate. Meanwhile, the government may need to set corresponding energy subsidy policies for people with poor socioeconomic conditions to facilitate their access to clean energy, thereby avoiding the long-term social impact of stunted growth in susceptible children.

Another interesting finding is that switching from solid fuels to clean fuels resulted in a reduction of stunting risk in children. Compared with those households using solid fuels throughout, children in those households switching to clean fuels had a 38% reduced risk of stunting, which was very closed to those children in households never using solid fuels. A cohort study of the Chinese population found that within 5 years after stopping the use solid fuels, the risk of death was significantly reduced (41). Similarly, it was reported that the prevalence of stunting in Bangladesh (42) decreased from 51 to 26% as their solid fuel use decreased from 92 to 82% over 10 years (2004–2014). However, there was no study continuously concerned about the longitudinal effect of switching to clean fuels on stunting risk. Therefore, we suggested prompting households to switch to clean energy source as early as possible, which might reduce the risk of stunting in children and thereby reduce the subsequent health and socioeconomic impacts resulting from growth retardation.

Unexpectedly, Cox regression showed that the association between coal use and the risk of childhood stunting was not significant during follow-up, although several studies have confirmed the effect of coal on the health status of children (43–45). We thought that it might be related to a much lower use of coal than biofuels in this study, and the inadequate sample size of coal use. Alternatively, researchers found that people tended to use biofuels for heating, which last longer than cooking, and thus the health-related effects of biofuels would be more obvious. However, given that no significant association between coal and stunting was found in this study, further studies are warranted to address this question.

Prior studies have shown that solid fuel might disturb children's growth mainly through direct influences from airborne particulate matter exposure and indirect influences from child morbidity (23). The combustion of solid fuels would produce harmful substances such as carbon monoxide, benzene, hydrogen cyanide, ammonia, nitrogen oxides, cresol and polycyclic aromatic hydrocarbons (PAHs), etc (46). Among these, carbon monoxide could bind to hemoglobin and lead to hypoxia in children, and benzene and cresol might cause their anemia (47). Children's central nervous system would also be adversely affected by the harmful compounds (21, 48–51). Additionally, PAHs were thought to be endocrine disruptors that cause growth retardation in children by affecting their endocrine system (52, 53). What's more, children exposed to HAP were strongly associated with respiratory diseases such as recurrent respiratory infections (54), resulting from oxidative stress and activation of inflammatory mediators triggered by the deposition of PM particles in the respiratory tract (18). It has also been suggested that air pollution might lead to vitamin D deficiency in children, thus affecting the bone growth (55). Therefore, we believe that solid fuel use could increases the risk of growth retardation in children through a comprehensive manner.

There are several strengths of this study including the large number of study population and the moderately long duration of follow-up for the evaluation of incident stunting. The sample size and length of follow-up ensured adequate number of stunting endpoints. Dynamic observation of household fuels use provided the opportunity to the see the impact of switching fuel type on stunting. A matching strategy was used to eliminate sample selection bias at baseline, which was neglected in previous studies.

Some limitations in this study should be recognized. First, we acknowledged the important effect of nutrition and exercise on children growth. However, only children aged between 10 and 15 years old provide the dietary diversity score and exercise data. We failed to further adjust for those confounders due to the small sample size. Second, information on current infection, immunization status and maternal exposure to pollutants during pregnancy was not available in the CFPS, though these factors were also considered to be associated with growth status in children. Third, housing ventilation and availability of a separate kitchen were also not measured due to the unavailability of such data in this investigation. Fourth, outdoor air pollution and other sources of pollution could not be estimated, potential residual confounding therefore could not be ruled out entirely in this study. However, we tried to adjust for the PM2.5 level in the sensitivity analysis. Fifth, information on solid fuel use was self-reported without validation, which might lead to misclassification of exposure. In addition, a certain number of participants in this study were lost to follow-up, though this is unavoidable in cohort study. Finally, the participants in this study were all from China, so the conclusions might need to prudently extend to other populations.

In summary, our study demonstrated the association between household solid fuel use and childhood stunting, figured out the underlying social factors that might lead to more solid fuel use and suggested that the switching from solid fuel use to clean fuel use could reduce the risk of childhood stunting. We suggested that the government should pay more attention to the availability of clean fuels for households, especially for those with low-income and low-educated levels, and strengthen the popularization and preferential policies for these households.

## Data availability statement

Publicly available datasets were analyzed in this study. This data can be found here: http://www.isss.pku.edu.cn/cfps.

## Ethics statement

The studies involving human participants were reviewed and approved by the Ethical Review Committee of Peking University. Written informed consent to participate in this study was provided by the participants' legal guardian/next of kin.

## Author contributions

LL and MYao contributed to conceptualization and writing–original draft. LL and MYan performed in revision. MYao contributed in data curation. MYan contributed to writing–review. FC and YW designed the research, obtained funding to support the study, supervision the research work, wrote, and revised the manuscript. LL, MYao, FC, and YW performed statistical analysis. All authors contributed meaningfully to this manuscript and approved the final version.

## Funding

This work was supported by the Research Grant of FC (No. 2022cffkyqdj) which was sponsored by the Second Affiliated Hospital of Chongqing Medical University, as well as the support from The First batch of key Disciplines On Public Health in Chongqing.

## Conflict of interest

The authors declare that the research was conducted in the absence of any commercial or financial relationships that could be construed as a potential conflict of interest.

## Publisher's note

All claims expressed in this article are solely those of the authors and do not necessarily represent those of their affiliated organizations, or those of the publisher, the editors and the reviewers. Any product that may be evaluated in this article, or claim that may be made by its manufacturer, is not guaranteed or endorsed by the publisher.
