# Supplementary material for: Household air pollution and childhood stunting in China: A prospective cohort study
Source: Front Public Health. 2022 Oct 21;10:985786. doi: 10.3389/fpubh.2022.985786 (PMC9650942; doi:10.3389/fpubh.2022.985786)

**Supplementary Materials**

**Table S1. The number and percentage of children with incident stunting in each wave**

|  | **Wave 1**  **(2010)** | **Wave 2**  **(2012)** | **Wave 3**  **(2014)** | **Wave 4**  **(2016)** | **Wave 5**  **(2018)** | **Total** |
| --- | --- | --- | --- | --- | --- | --- |
| **N** | 772 | 772 | 618 | 554 | 534 | 772 |
| **Stunting** |  |  |  |  |  |  |
| No | 772  (100.00%) | 618  (80.05%) | 554  (89.64%) | 534  (96.39%) | 524  (98.13%) | 524  (67.88%) |
| Yes | 0  (0%) | 154  (19.95%) | 64  (10.36%) | 20  (3.61%) | 10  (1.87%) | 248  (32.12%) |

**Table S2.** **Baseline characteristics of children grouped by fuel use type at baseline in the longitudinal analysis**

| Characteristics | Total | Clean fuel | Solid fuel | P-value |
| --- | --- | --- | --- | --- |
|  | N=772 | N=415 | N=357 |  |
| Age (years) | 4.00 [2.75;6.00] | 4.00 [2.00;6.00] | 4.00 [3.00;6.00] | 0.385 |
| Gender: |  |  |  | 1.000 |
| Boy | 423 (54.8%) | 227 (54.7%) | 196 (54.9%) |  |
| Girl | 349 (45.2%) | 188 (45.3%) | 161 (45.1%) |  |
| Paternal height (meters) | 1.70 [1.68;1.75] | 1.72 [1.69;1.75] | 1.70 [1.68;1.75] | 0.012 |
| Maternal height (meters) | 1.60 [1.58;1.64] | 1.60 [1.58;1.64] | 1.60 [1.57;1.63] | 0.183 |
| Paternal education: |  |  |  | <0.001 |
| Under primary school | 190 (24.6%) | 61 (14.7%) | 129 (36.1%) |  |
| Above primary school | 582 (75.4%) | 354 (85.3%) | 228 (63.9%) |  |
| Maternal education: |  |  |  | <0.001 |
| Under primary school | 244 (31.6%) | 78 (18.8%) | 166 (46.5%) |  |
| Above primary school | 528 (68.4%) | 337 (81.2%) | 191 (53.5%) |  |
| Residence: |  |  |  | <0.001 |
| Urban | 357 (46.2%) | 263 (63.4%) | 94 (26.3%) |  |
| Rural | 415 (53.8%) | 152 (36.6%) | 263 (73.7%) |  |
| Household income: |  |  |  | <0.001 |
| Below 10,000 yuan/year | 64 (8.29%) | 18 (4.34%) | 46 (12.9%) |  |
| Above 10,000 yuan/year | 708 (91.7%) | 397 (95.7%) | 311 (87.1%) |  |
| Siblings: |  |  |  | <0.001 |
| 1 | 425 (55.1%) | 264 (63.6%) | 161 (45.1%) |  |
| 2 | 264 (34.2%) | 126 (30.4%) | 138 (38.7%) |  |
| >=3 | 83 (10.8%) | 25 (6.02%) | 58 (16.2%) |  |
| Cooking water: |  |  |  | <0.001 |
| Tap/purified water | 463 (60.0%) | 331 (79.8%) | 132 (37.0%) |  |
| Surface-exposed/well water | 309 (40.0%) | 84 (20.2%) | 225 (63.0%) |  |

All data are expressed as median [Q1-Q3] or proportion in %; All comparison was conducted between subjects with different fuel use type by using either the Student’s t-test for continuous data or the Chi-square test for categorical data.**Table S3. Baseline characteristics between children included and excluded in the cross-sectional analysis**

| Characteristics | Total | Excluded Sample | Analytic Sample | P-value |
| --- | --- | --- | --- | --- |
|  | N=7025 | N=1012 | N=6013 |  |
| Household fuel use: |  |  |  | <0.001 |
| Solid fuels | 3853 (54.8%) | 703 (69.5%) | 3150 (52.4%) |  |
| Clean fuels | 3172 (45.2%) | 309 (30.5%) | 2863 (47.6%) |  |
| Age (years) | 7.00 [3.00;11.00] | 3.00 [1.00;6.00] | 8.00 [4.00;12.00] | <0.001 |
| Gender: |  |  |  | 0.005 |
| Boy | 3728 (53.1%) | 579 (57.2%) | 3149 (52.4%) |  |
| Girl | 3297 (46.9%) | 433 (42.8%) | 2864 (47.6%) |  |
| Paternal height (meters) | 1.70 [1.65;1.73] | 1.70 [1.65;1.73] | 1.70 [1.66;1.73] | 0.002 |
| Maternal height (meters) | 1.60 [1.56;1.63] | 1.60 [1.55;1.62] | 1.60 [1.56;1.63] | 0.045 |
| Paternal education: |  |  |  | <0.001 |
| Under primary school | 2858 (40.7%) | 521 (51.5%) | 2337 (38.9%) |  |
| Above primary school | 4167 (59.3%) | 491 (48.5%) | 3676 (61.1%) |  |
| Maternal education: |  |  |  | <0.001 |
| Under primary school | 3699 (52.7%) | 633 (62.5%) | 3066 (51.0%) |  |
| Above primary school | 3326 (47.3%) | 379 (37.5%) | 2947 (49.0%) |  |
| Residence: |  |  |  | <0.001 |
| Urban | 2653 (37.8%) | 235 (23.2%) | 2418 (40.2%) |  |
| Rural | 4372 (62.2%) | 777 (76.8%) | 3595 (59.8%) |  |
| Household income: |  |  |  | <0.001 |
| Below 10,000 yuan/year | 1142 (16.3%) | 248 (24.5%) | 894 (14.9%) |  |
| Above 10,000 yuan/year | 5883 (83.7%) | 764 (75.5%) | 5119 (85.1%) |  |
| Siblings: |  |  |  | <0.001 |
| 1 | 3209 (45.7%) | 329 (32.5%) | 2880 (47.9%) |  |
| 2 | 2785 (39.6%) | 464 (45.8%) | 2321 (38.6%) |  |
| >=3 | 1031 (14.7%) | 219 (21.6%) | 812 (13.5%) |  |
| Cooking water: |  |  |  | <0.001 |
| Tap/purified water | 3577 (50.9%) | 362 (35.8%) | 3215 (53.5%) |  |
| Surface-exposed/well water | 3448 (49.1%) | 650 (64.2%) | 2798 (46.5%) |  |

All data are expressed as median [Q1-Q3] or proportion in %; All comparison was conducted between subjects with different fuel use type by using either the Student’s t-test for continuous data or the Chi-square test for categorical data.

**Table S4. Baseline characteristics between children included and excluded in the longitudinal analysis**

| Characteristics | Total | Excluded Sample | Analytic Sample | P-value |
| --- | --- | --- | --- | --- |
|  | N=6013 | N=5241 | N=772 |  |
| Household fuel use: |  |  |  | <0.001 |
| Solid fuels | 3150 (52.4%) | 2793 (53.3%) | 357 (46.2%) |  |
| Clean fuels | 2863 (47.6%) | 2448 (46.7%) | 415 (53.8%) |  |
| Age (years) | 8.00 [4.00;12.00] | 9.00 [5.00;12.00] | 4.00 [2.75;6.00] | <0.001 |
| Gender: |  |  |  | 0.160 |
| Boy | 3149 (52.4%) | 2726 (52.0%) | 423 (54.8%) |  |
| Girl | 2864 (47.6%) | 2515 (48.0%) | 349 (45.2%) |  |
| Paternal height (meters) | 1.70 [1.66;1.73] | 1.70 [1.65;1.73] | 1.70 [1.68;1.75] | <0.001 |
| Maternal height (meters) | 1.60 [1.56;1.63] | 1.60 [1.55;1.62] | 1.60 [1.58;1.64] | <0.001 |
| Paternal education: |  |  |  | <0.001 |
| Under primary school | 2337 (38.9%) | 2147 (41.0%) | 190 (24.6%) |  |
| Above primary school | 3676 (61.1%) | 3094 (59.0%) | 582 (75.4%) |  |
| Maternal education: |  |  |  | <0.001 |
| Under primary school | 3066 (51.0%) | 2822 (53.8%) | 244 (31.6%) |  |
| Above primary school | 2947 (49.0%) | 2419 (46.2%) | 528 (68.4%) |  |
| Residence: |  |  |  | <0.001 |
| Urban | 2418 (40.2%) | 2061 (39.3%) | 357 (46.2%) |  |
| Rural | 3595 (59.8%) | 3180 (60.7%) | 415 (53.8%) |  |
| Household income: |  |  |  | <0.001 |
| Below 10,000 yuan/year | 894 (14.9%) | 830 (15.8%) | 64 (8.29%) |  |
| Above 10,000 yuan/year | 5119 (85.1%) | 4411 (84.2%) | 708 (91.7%) |  |
| Siblings: |  |  |  | <0.001 |
| 1 | 2880 (47.9%) | 2455 (46.8%) | 425 (55.1%) |  |
| 2 | 2321 (38.6%) | 2057 (39.2%) | 264 (34.2%) |  |
| >=3 | 812 (13.5%) | 729 (13.9%) | 83 (10.8%) |  |
| Cooking water: |  |  |  | <0.001 |
| Tap/purified water | 3215 (53.5%) | 2752 (52.5%) | 463 (60.0%) |  |
| Surface-exposed/well water | 2798 (46.5%) | 2489 (47.5%) | 309 (40.0%) |  |

All data are expressed as median [Q1-Q3] or proportion in %; All comparison was conducted between subjects with different fuel use type by using either the Student’s t-test for continuous data or the Chi-square test for categorical data.**Table S5. The association between solid fuel use and severe stunting at baseline using unmatched and matched data**

|  | **Unmatched data** | **Matched data** |
| --- | --- | --- |
|  | OR (95% CI) | OR (95% CI) |
| **Household fuel use** | | |
| Clean fuels | 1.00 (ref) | 1.00 (ref) |
| Solid fuels | 1.40 (1.20,1.63) ^***^ | 1.40 (1.19,1.65) ^***^ |
| **Types of household fuel use** | | |
| Clean fuels | 1.00 (ref) | 1.00 (ref) |
| Coal | 1.31 (1.06,1.63) ^*^ | 1.28 (1.00,1.65) |
| Firewood/straw | 1.43 (1.22,1.68) ^***^ | 1.44 (1.21,1.72) ^***^ |

**Notes:** All the models were adjusted for age, gender, parental education, parental height, household income, residence, siblings and cooking water; *p < 0.05; **p < 0.01; ***p < 0.001.

**Table S6. Covariates balancing after propensity score matching**

| **Variable** | **Mean** | | **Bias (%)** | **Bias reduction (%)** | **t** | **p-value** |
| --- | --- | --- | --- | --- | --- | --- |
|  | Treated | Control |  |  |  |  |
| Age | 8.07 | 7.83 | 5.6 | -74.1 | 2.21 | 0.03 |
| Gender | 0.52 | 0.54 | -3.0 | -161.3 | -1.19 | 0.23 |
| Paternal height | 1.69 | 1.69 | -2.0 | 93.6 | -0.77 | 0.44 |
| Maternal height | 1.59 | 1.59 | 1.6 | 86.5 | 0.62 | 0.54 |
| Paternal education | 0.51 | 0.50 | 1.2 | 97.8 | 0.46 | 0.65 |
| Maternal education | 0.66 | 0.66 | 0.3 | 99.5 | 0.12 | 0.90 |
| Siblings | 1.81 | 1.79 | 2.6 | 94.7 | 1.01 | 0.31 |
| Household income | 0.20 | 0.18 | 5.0 | 85.2 | 1.77 | 0.08 |
| Cooking water | 0.34 | 0.33 | 0.8 | 99.2 | 0.29 | 0.78 |
| Urban | 0.20 | 0.19 | 2.8 | 97.1 | 1.23 | 0.22 |
| **Sample** | **Ps R2** | **LR chi2** | **p>chi2** | **Mean Bias** | **R** | **Var (%)** |
| Unmatched | 0.247 | 2059.06 | 0 | 44.7 | 0.8 | 75 |
| Matched | 0.002 | 13.44 | 0.2 | 2.5 | 1.02 | 25 |

**Table S7.** The odds ratio of solid fuel use on stunting in sub-group analysis according to the residence.

|  | **Unmatched Model** | | **Matched Model** | |
| --- | --- | --- | --- | --- |
|  | Urban area | Rural area | Urban area | Rural area |
|  | OR (95%CI) | OR (95%CI) | OR (95%CI) | OR (95%CI) |
| **Household fuel use** | | | | |
| Clean fuels | 1.00 (ref) | 1.00 (ref) | 1.00 (ref) | 1.00 (ref) |
| Solid fuels | 1.53  (1.20,1.94) ^***^ | 1.34  (1.14,1.58) ^***^ | 1.59  (1.22,2.06) ^***^ | 1.33  (1.12,1.59) ^**^ |
| **Types of household fuel use** | | | | |
| Clean fuels | 1.00 (ref) | 1.00 (ref) | 1.00 (ref) | 1.00 (ref) |
| Coal | 1.41  (1.01,1.96) ^*^ | 1.20  (0.94,1.53) | 1.50  (1.06,2.13) ^*^ | 1.15  (0.85,1.55) |
| Firewood/straw | 1.62  (1.21,2.18) ^**^ | 1.38  (1.17,1.63) ^***^ | 1.65  (1.20,2.26) ^**^ | 1.38  (1.14,1.66) ^***^ |

**Notes:** All the models were adjusted for age, gender, parental education, parental height, household income, siblings and cooking water; *p < 0.05; **p < 0.01; ***p < 0.001.

**Table S8. Sensitivity analysis in the cross-sectional analysis**

|  | Model 0 | Model 1 | Model 2 | Model 3 |
| --- | --- | --- | --- | --- |
|  | OR (95%CI) | OR (95%CI) | OR (95%CI) | OR (95%CI) |
| Household fuel use | | | | |
| Clean fuels | 1.00 (ref) | 1.00 (ref) | 1.00 (ref) | 1.00 (ref) |
| Solid fuels | 1.42 (1.24,1.63)  ^***^ | 1.28  (1.11,1.48)  ^***^ | 1.33  (1.15,1.54)  ^***^ | 1.23  (1.06,1.43)  ^**^ |
| Types of household fuel use | | | | |
| Clean fuels | 1.00 (ref) | 1.00 (ref) | 1.00 (ref) | 1.00(ref) |
| Coal | 1.30 (1.07,1.58)  ^**^ | 1.14  (0.93,1.40) | 1.30  (1.06,1.60)  ^*^ | 1.14  (0.92,1.41) |
| Firewood/straw | 1.47 (1.27,1.70)  ^***^ | 1.34  (1.14,1.56)  ^***^ | 1.34  (1.15,1.57)  ^***^ | 1.27  (1.08,1.50)  ^**^ |
| N | 6013 | 6,013 | 5,259 | 5259 |

**Notes:** Model 0: adjusted for age, gender, parental education, parental height, household income, siblings and cooking water Model 1: Model 0 plus adjustment for PM_2.5_ and province dummy; Model 2: Model 0 plus adjustment for birth weight and breastfeeding; Model 3: Model 0 plus adjustment for PM_2.5_, province dummy, birth weight, breastfeeding. *p < 0.05; **p < 0.01; ***p < 0.001.

**Table S9. Sensitivity analysis by excluding children with HAZ score beyond ±6SD**

|  | **Unmatched Model** | **Matched Model** |
| --- | --- | --- |
|  | OR (95% CI) | OR (95% CI) |
| **Household fuel use** | | |
| Clean fuels | 1.00 (ref) | 1.00 (ref) |
| Solid fuels | 1.37  (1.19,1.57) ^***^ | 1.28  (1.10,1.48) ^**^ |
| **Types of household fuel use** | | |
| Clean fuels | 1.00 (ref) | 1.00 (ref) |
| Coal | 1.25  (1.03,1.53) ^*^ | 1.20  (0.96,1.50) |
| Firewood/straw | 1.41  (1.21,1.64) ^***^ | 1.31  (1.11,1.54) ^**^ |

**Notes:** All the models were adjusted for age, gender, parental education, parental height, household income, residence, siblings and cooking water; *p < 0.05; **p < 0.01; ***p < 0.001.

**Table S10. Proportional hazard assumption for Cox model for association between household fuel use and childhood stunting using Log-rank test and Schoenfeld's test**

|  | **p value for Log-rank test** | **P value for Schoenfeld's test** |
| --- | --- | --- |
| **Household fuel use (*vs* clean fuels use)** |  |  |
| Solid fuels | <0.0001 | 0.9415 |
| **Duration of** **solid fuel use (*vs* 0)** |  |  |
| 1-7 years | <0.0001 | 0.8615 |
| ≥8 years |  | 0.7190 |
| **Types of solid fuel use (*vs* clean fuels use)** |  |  |
| Coal | <0.0001 | 0.5934 |
| Firewood/straw |  | 0.8797 |

**Table S11. Hazard ratios of solid fuel use for the future risk of stunting stratified by the residence in the longitudinal analysis**

| Follow-up period | 2010→2014 | | | 2010→2018 | | |
| --- | --- | --- | --- | --- | --- | --- |
|  | Urban area | | Rural area | Urban area | Rural area | |
|  | HR (95%CI) | | HR (95%CI) | HR (95%CI) | HR (95%CI) | |
| Household fuel use | | | | | | |
| Clean fuels | 1.00 (ref) | 1.00 (ref) | | 1.00 (ref) | | 1.00 (ref) |
| Solid fuels | 1.11  (0.72,1.72) | 1.47  (1.17,1.84)  ^***^ | | 0.92  (0.53,1.63) | | 1.66  (1.26,2.19)  ^***^ |
| Types of household fuel use | | | | | | |
| Clean fuels | 1.00 (ref) | 1.00 (ref) | | 1.00 (ref) | | 1.00 (ref) |
| Coal | 0.93  (0.43,2.01) | 1.59  (1.05,2.40)  ^*^ | | 0.52  (0.16,1.75) | | 1.66  (1.01,2.73)  ^*^ |
| Firewood/straw | 1.19  (0.72,1.95) | 1.45  (1.15,1.83)  ^**^ | | 1.13  (0.62,2.06) | | 1.66  (1.25,2.21)  ^***^ |
| Duration of solid fuel use | | | | | | |
| 0 | 1.00 (ref) | 1.00 (ref) | | 1.00 (ref) | | 1.00 (ref) |
| 1-3 years | 0.88  (0.54,1.43) | 1.51  (1.08,2.11)  ^*^ | |  | |  |
| >=4 years | 1.28  (0.74,2.21) | 1.78  (1.28,2.48)  ^***^ | |  | |  |
| 1-7 years |  |  | | 1.09  (0.64,1.86) | | 1.20  (0.81,1.77) |
| >=8 years |  |  | | 0.78  (0.29,2.05) | | 1.93  (1.28,2.90)  ^**^ |

**Notes:** All the models were adjusted for age, gender, parental education, parental height, household income, siblings and cooking water; *p < 0.05; **p < 0.01; ***p < 0.001.

**Table S12. Sensitivity analysis in the longitudinal analysis**

| Follow-up period |  | Model 0 | Model 1 | Model 2 | Model 3 |
| --- | --- | --- | --- | --- | --- |
|  |  | HR (95%CI) | HR (95%CI) | HR (95%CI) | HR (95%CI) |
| 2010→2014 |  |  |  |  |  |
|  | Household fuel use | | | | |
|  | Clean fuels | 1.00 (ref) | 1.00 (ref) | 1.00 (ref) | 1.00 (ref) |
|  | Solid fuels | 1.40  (1.15,1.71)  ^***^ | 1.37  (1.12,1.67)  ^**^ | 1.36  (1.11,1.68)  ^**^ | 1.33  (1.08,1.64)  ^**^ |
|  | Types of household fuel use | | | | |
|  | Clean fuels | 1.00 (ref) | 1.00 (ref) | 1.00 (ref) | 1.00 (ref) |
|  | Coal | 1.37  (0.96,1.97) | 1.28  (0.89,1.84) | 1.37  (0.95,1.99) | 1.28  (0.88,1.87) |
|  | Firewood/straw | 1.41  (1.14,1.73)  ^**^ | 1.39  (1.12,1.72)  ^**^ | 1.36  (1.09,1.69)  ^**^ | 1.35  (1.08,1.68)  ^**^ |
|  | Duration of solid fuel use | | | | |
|  | 0 | 1.00 (ref) | 1.00 (ref) | 1.00 (ref) | 1.00 (ref) |
|  | 1-3 years | 1.30  (1.00,1.69)  ^*^ | 1.24  (0.95,1.62) | 1.25  (0.96,1.64) | 1.16  (0.88,1.53) |
|  | >=4 years | 1.60  (1.23,2.10)  ^***^ | 1.52  (1.16,1.99)  ^**^ | 1.56  (1.18,2.07)  ^**^ | 1.47  (1.10,1.95)  ^**^ |
|  | N | 1789 | 1789 | 1684 | 1684 |
| 2010→2018 |  |  |  |  |  |
|  | Household fuel use | | | | |
|  | Clean fuels | 1.00 (ref) | 1.00 (ref) | 1.00 (ref) | 1.00 (ref) |
|  | Solid fuels | 1.46  (1.15,1.86)  ^**^ | 1.47  (1.15,1.87)  ^**^ | 1.45  (1.14,1.85)  ^**^ | 1.45  (1.13,1.85)  ** |
|  | Types of household fuel use | | | | |
|  | Clean fuels | 1.00 (ref) | 1.00 (ref) | 1.00 (ref) | 1.00 (ref) |
|  | Coal | 1.24  (0.79,1.94) | 1.17  (0.75,1.84) | 1.19  (0.75,1.89) | 1.10  (0.69,1.76) |
|  | Firewood/straw | 1.52  (1.18,1.96)  ** | 1.57  (1.20,2.04)  ^***^ | 1.52  (1.18,1.97)  ^**^ | 1.57  (1.20,2.05)  ^**^ |
|  | Duration of solid fuel use | | | | |
|  | 0 | 1.00 (ref) | 1.00 (ref) | 1.00 (ref) | 1.00 (ref) |
|  | 1-7 years | 1.20  (0.88,1.63) | 1.17  (0.86,1.61) | 1.20  (0.87,1.64) | 1.15  (0.83,1.60) |
|  | >=8 years | 1.77  (1.25,2.52)  ** | 1.74  (1.20,2.52)  ^**^ | 1.76  (1.24,2.51)  ^**^ | 1.73  (1.19,2.52)  ^**^ |
|  | N | 772 | 772 | 763 | 763 |

**Notes:** Model 0: adjusted for age, gender, parental education, parental height, household income, residence, siblings and cooking water. Model 1: Model 0 plus adjustment for PM_2.5_ and Province dummy; Model 2: Model 0 plus adjustment for birth weight and breastfeeding; Model 3: Model 0 plus adjustment for PM_2.5_, Province dummy, birth weight, breastfeeding. *p < 0.05; **p < 0.01; ***p < 0.001.

**Table S13. Sensitivity analysis after excluding children with HAZ score beyond ±6SD in the longitudinal analysis**

| Follow-up period |  | Model 0 | Model 1 | Model 2 | Model 3 |
| --- | --- | --- | --- | --- | --- |
|  |  | HR (95%CI) | HR (95%CI) | HR (95%CI) | HR (95%CI) |
| 2010→2014 |  | | | | |
|  | Household fuel use | | | | |
|  | Clean fuels | 1.00 (ref) | 1.00 (ref) | 1.00 (ref) | 1.00 (ref) |
|  | Solid fuels | 2.01  (1.66,2.44)  ^***^ | 2.04  (1.69,2.47)  ^***^ | 1.65  (1.35,2.03)  ^***^ | 1.41  (1.14,1.73)  ^**^ |
|  | Types of household fuel use | | | | |
|  | Clean fuels | 1.00 (ref) | 1.00 (ref) | 1.00 (ref) | 1.00 (ref) |
|  | Coal | 1.69  (1.18,2.41)  ^**^ | 1.66  (1.16,2.38)  ^**^ | 1.49  (1.04,2.14)  ^*^ | 1.43  (1.00,2.06) |
|  | Firewood/straw | 2.10  (1.71,2.57)  ^***^ | 2.15  (1.76,2.62)  ^***^ | 1.70  (1.37,2.11)  ^***^ | 1.40  (1.12,1.74)  ^**^ |
|  | Duration of solid fuel use | | | | |
|  | 0 | 1.00 (ref) | 1.00 (ref) | 1.00 (ref) | 1.00 (ref) |
|  | 1-3 years | 1.77  (1.38,2.28)  ^***^ | 1.88  (1.47,2.41)  ^***^ | 1.54  (1.18,2.00)  ^**^ | 1.28  (0.98,1.68) |
|  | >=4 years | 2.59  (2.04,3.28)  ^***^ | 2.69  (2.13,3.40)  ^***^ | 2.05  (1.58,2.66)  ^***^ | 1.60  (1.21,2.11)  ^***^ |
| 2010→2018 |  | | | | |
|  | Household fuel use | | | | |
|  | Clean fuels | 1.00 (ref) | 1.00 (ref) | 1.00 (ref) | 1.00 (ref) |
|  | Solid fuels | 2.00  (1.58,2.52)  ^***^ | 2.02  (1.60,2.55)  ^***^ | 1.78  (1.39,2.27)  ^***^ | 1.43  (1.11,1.83)  ^**^ |
|  | Types of household fuel use | | | | |
|  | Clean fuels | 1.00 (ref) | 1.00 (ref) | 1.00 (ref) | 1.00 (ref) |
|  | Coal | 1.46  (0.92,2.34) | 1.43  (0.89,2.30) | 1.37  (0.85,2.19) | 1.23  (0.78,1.95) |
|  | Firewood/straw | 2.14  (1.68,2.74)  ^***^ | 2.18  (1.72,2.78)  ^***^ | 1.89  (1.46,2.45)  ^***^ | 1.48  (1.14,1.93)  ^**^ |
|  | Duration of solid fuel use | | | | |
|  | 0 | 1.00 (ref) | 1.00 (ref) | 1.00 (ref) | 1.00 (ref) |
|  | 1-7 years | 1.66  (1.26,2.19)  ^***^ | 1.75  (1.33,2.31)  ^***^ | 1.51  (1.12,2.04)  ^**^ | 1.19  0.87,1.63 |
|  | >=8 years | 2.88  (2.10,3.95)  ^***^ | 2.91  (2.13,3.97)  ^***^ | 2.45  (1.75,3.44)  ^***^ | 1.68  (1.16,2.42)  ^**^ |

**Notes:** Model 0: unadjusted; Model 1: adjusted for age and gender; Model 2: model 1 plus adjustment for parental education, parental height; Model 3: model 2 plus adjustment for household income, residence, siblings, cooking water. *p < 0.05; **p < 0.01; ***p < 0.001.

Abbreviation: HAZ, height-for-age z-score.

**Table S14.** **The effect of switching time of household fuel use on childhood stunting**

| Switching year | Model 0 | Model 1 | Model 2 | Model 3 |
| --- | --- | --- | --- | --- |
|  | OR (95%CI) | OR (95%CI) | OR (95%CI) | OR (95%CI) |
| No switching | 1.00 (ref) | 1.00 (ref) | 1.00 (ref) | 1.00 (ref) |
| switching in 2018 | 0.66  (0.40,1.11) | 0.66  (0.39,1.11) | 0.67  (0.40,1.13) | 0.72  (0.43,1.23) |
| switching in 2016 | 0.54  (0.34,0.86) ^**^ | 0.54  (0.34,0.86) ^**^ | 0.58  (0.36,0.93) ^*^ | 0.62  (0.39,1.01) |
| switching in 2014 | 0.34  (0.21,0.54) ^***^ | 0.35  (0.22,0.56) ^***^ | 0.35  (0.22,0.58) ^***^ | 0.39  (0.24,0.64) ^***^ |
| switching in 2012 | 0.40  (0.28,0.58) ^***^ | 0.41  (0.28,0.59) ^***^ | 0.42  (0.29,0.61) ^***^ | 0.45  (0.31,0.66) ^***^ |
| Continuous (per two years) | 0.78  (0.71,0.85) ^***^ | 0.78  (0.71,0.86) ^***^ | 0.79  (0.72,0.87) ^***^ | 0.80  (0.73,0.88) ^***^ |

**Notes:** Model 0: unadjusted; Model 1: adjusted for age and gender; Model 2: model 1 plus adjustment for parental education, parental height; Model 3: model 2 plus adjustment for household income, residence, siblings, cooking water. *p < 0.05; **p < 0.01; ***p < 0.001.

**Figure S1.** **Flowchart of participant selection for this study**

**
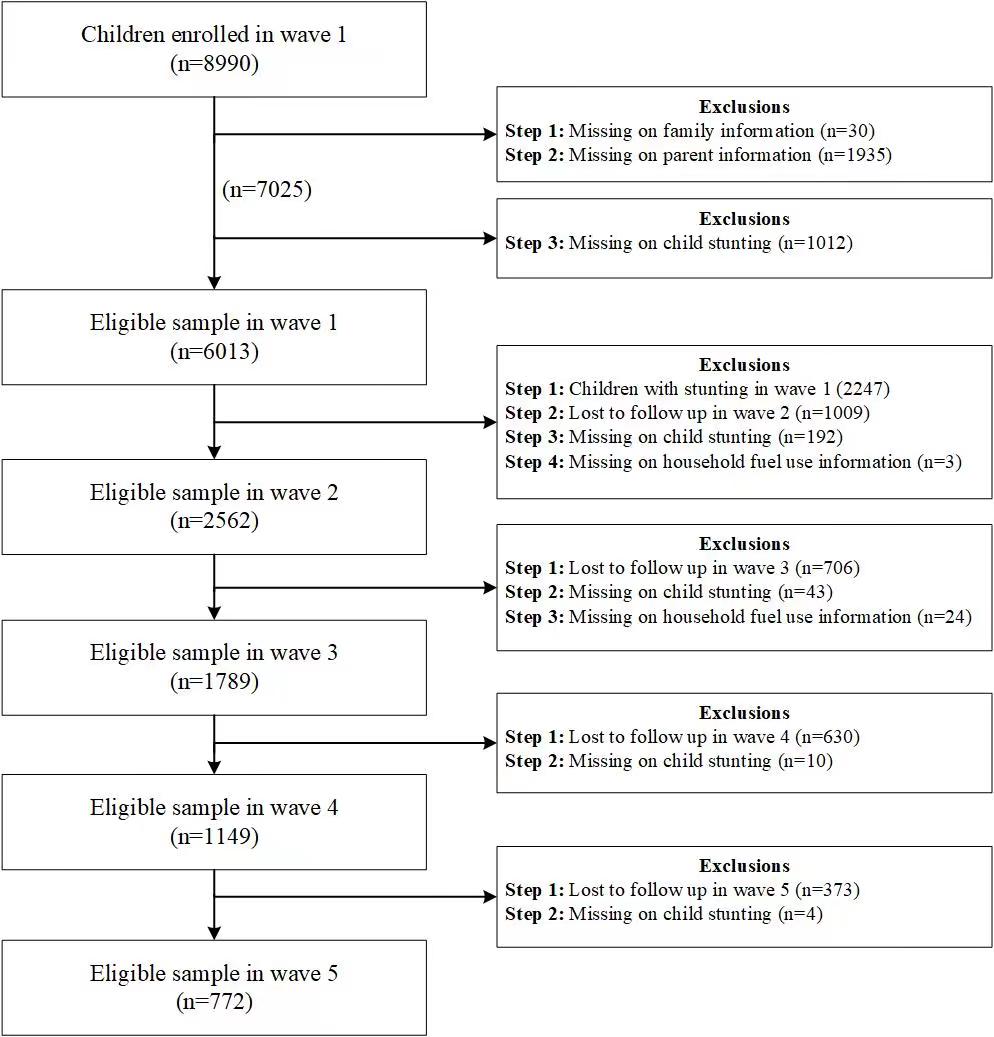
**

**Figure S2. China Family Panel Surveys sampling distribution map**

**
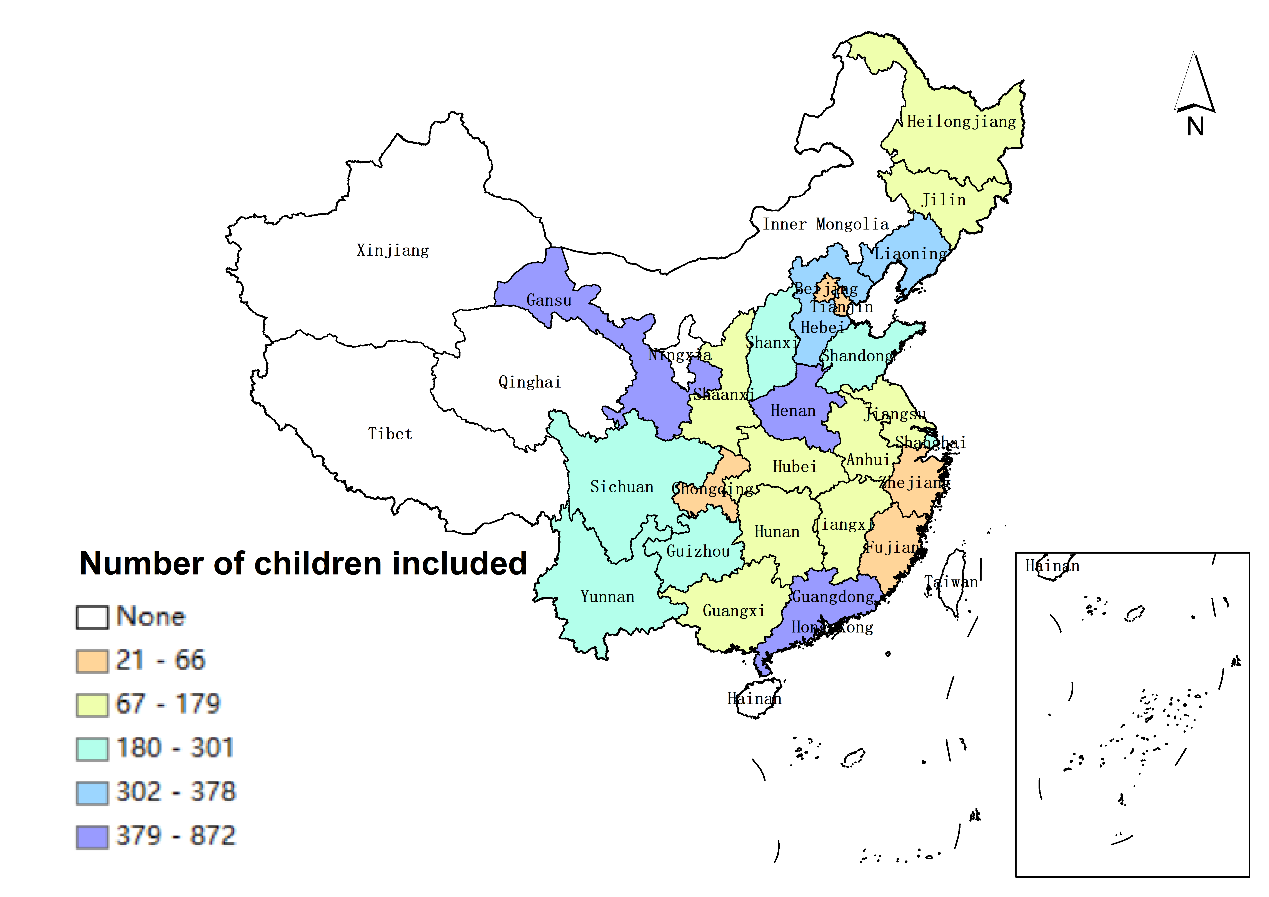
**

**Figure S3. Mediation analysis diagram**


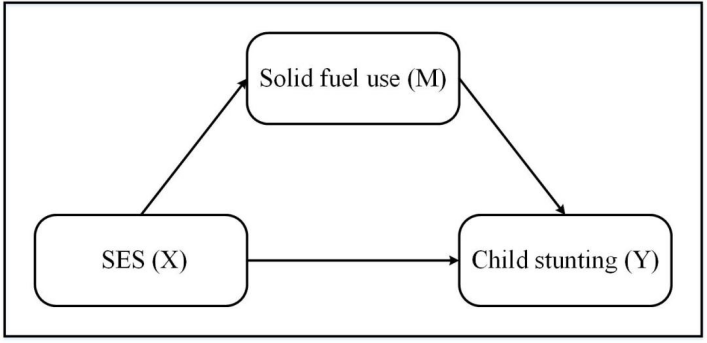


**Figure S4.** **Test of common support for Propensity Score Matching**


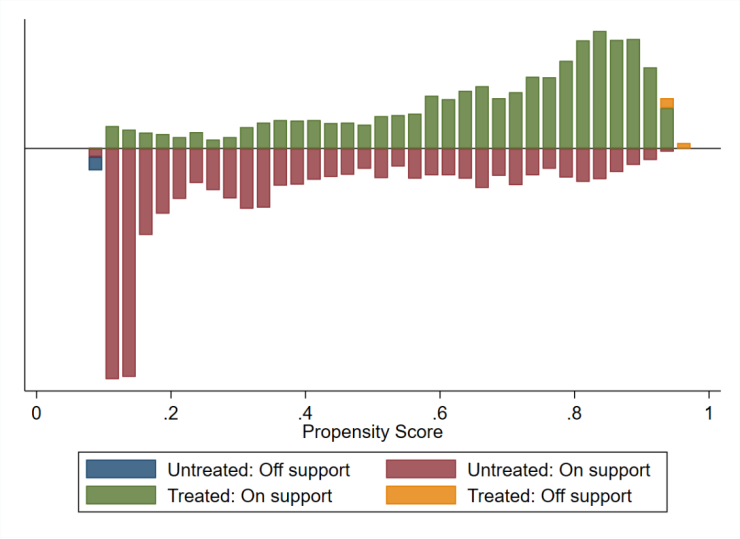


**Figure S5.** **Density Plot for Propensity Score**


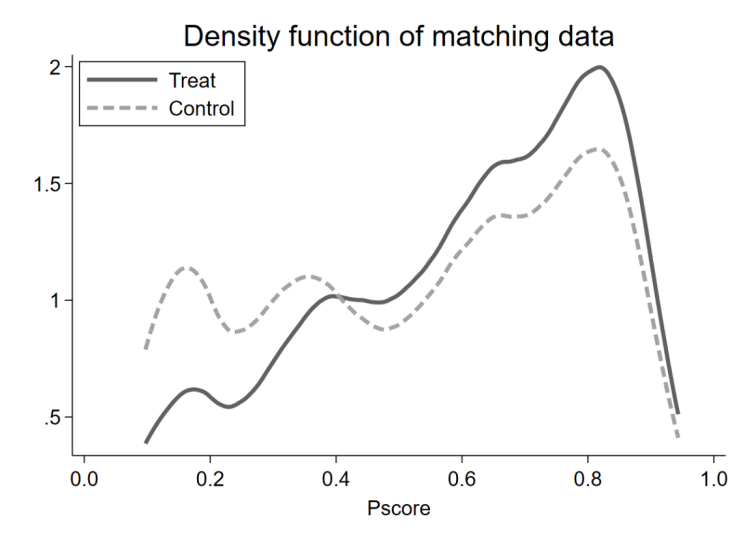


**Figure S6. Kaplan-Meier plot of Cox model examining the association between household fuel use and childhood stunting**


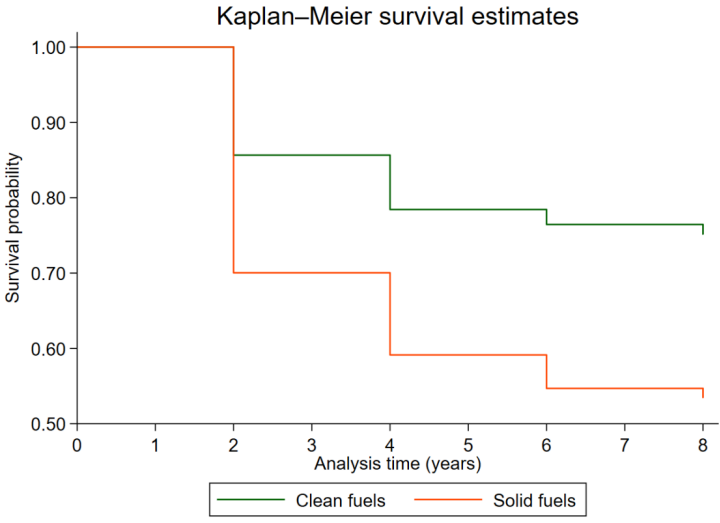


Figure S7. The directed acyclic graph on the selection of covariates


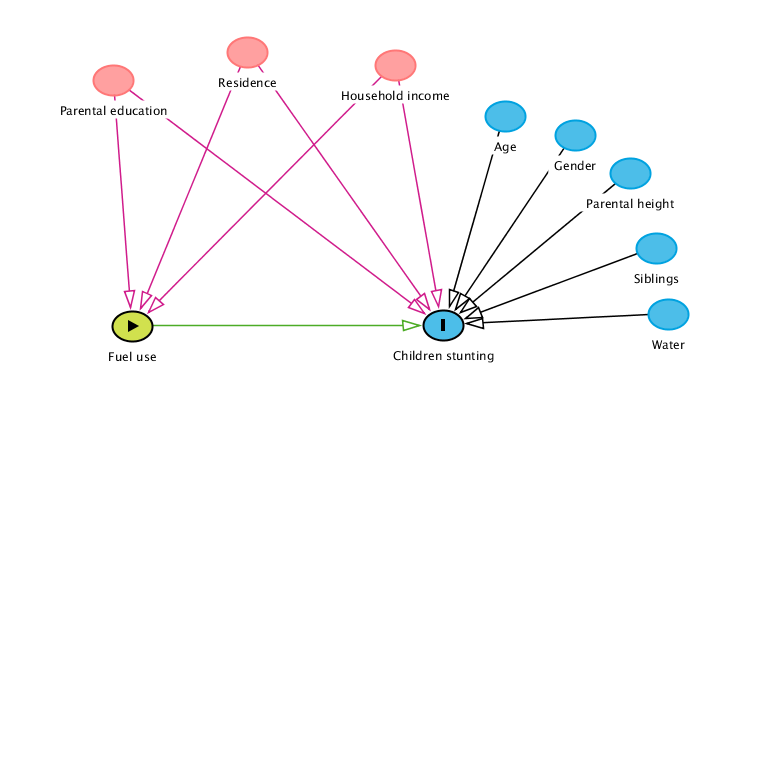

Supplement: Supplementary file 1 [file Data_Sheet_1.DOCX]
